# Acoustic Emission-Based Offshore Pipeline Valve Leakage Detection Toward Enhanced Process Safety

**DOI:** 10.3390/s26144451

**Published:** 2026-07-13

**Authors:** Hongdong Qin, Xingshuang Hao, Zhenhao Zhu, Weizhe Ren, Xiaolong Qiu, Yuchen Lu, Hongbing Liu, Yuxuan Zhang

**Affiliations:** 1 Yantai Research Institute, Harbin Engineering University, Yantai 264006, China; 2 College of Intelligent Science and Engineering, Beijing University of Agriculture, Beijing 102206, China; 3 Department of Computer and Electrical Engineering, Mid Sweden University, 85170 Sundsvall, Sweden

**Keywords:** marine pipeline leak detection, acoustic emission, lightweight, parameter-free attention, deep learning

## Abstract

Valve leakage in marine oil and gas pipelines is a critical failure mode that threatens operational safety, ecological integrity and production economic benefits, creating an urgent demand for accurate, real-time and robust fault diagnosis systems. Acoustic Emission (AE) technology captures transient acoustic signatures generated by leakage to enable non-intrusive online monitoring, while deep learning supports intelligent analysis through automatic signal feature extraction. Nevertheless, traditional AE-based leakage diagnosis methods rely heavily on manual feature engineering and fixed signal processing rules. Existing AE-driven deep learning methods fail to simultaneously deliver high detection accuracy, low inference latency and strong noise immunity, hindering their practical deployment on offshore platforms. To address these limitations, this paper proposes a Parameter-free Star-shaped Attention Fusion Network (SAFNet) for lightweight valve leakage localization using AE signals. Centered on the Temporal Pyramid Encoder (TPE) and Progressive Lightweight Star-shaped Attention (PLSA) module, SAFNet integrates Dual Bilinear Star Mapping (DBSM), Energy-Driven Feature Refiner (EDFR) and Multi-Scale Gated Attention Fusion (MS-GAF) modules. This architecture achieves efficient multi-scale temporal feature extraction, parameter-free nonlinear enhancement, noise-resistant refined feature processing and adaptive hierarchical feature fusion. The proposed method is applicable to valve leakage diagnosis of marine oil and gas pipelines under variable pressure and complex marine noise conditions. Comprehensive experiments are conducted on a dataset constructed by combining laboratory controlled leakage signals with real marine background noise recorded from the Liwan 3-1 offshore platform. The experimental results reveal that SAFNet balances high detection accuracy, compact model size and low inference latency simultaneously. Specifically, the network maintains a stable detection accuracy above 95% under pipeline pressures ranging from 2 MPa to 5 MPa, and exhibits excellent stability under extreme heavy noise environments. Ablation experiments further validate the synergistic performance gain brought by all core modules. The presented network delivers an efficient lightweight solution for valve leakage localization under simulated marine acoustic conditions, promotes the development of intelligent monitoring technologies for marine pipeline systems, and comprehensively improves offshore operational safety and marine ecological protection capacity.

## 1. Introduction

Offshore oil and gas pipelines are critical infrastructure for offshore production systems. However, any failure in their integrity poses a serious threat to the safety of offshore industrial processes and environmental protection [1,2,3,4]. Pipeline leaks and valve failures can lead to catastrophic consequences, including fires, explosions, and the release of toxic substances [5,6,7], posing a serious threat to the continued safe operation of offshore platforms and the entire offshore production system [8,9]. Traditional periodic manual inspections are hampered by extreme operating conditions and high maintenance costs; they cannot provide real-time insights into pipeline health and struggle to meet the engineering requirements of condition-based maintenance. Therefore, structural health monitoring technology is not only a key means of ensuring the stable operation of offshore oil and gas platforms, but also an indispensable safeguard for process safety in the offshore industry [10,11]. Non-destructive testing (NDT) technology serves as a critical technical foundation for structural health monitoring. Among these, acoustic emission (AE) technology [12,13,14,15,16] with its technical advantages of being non-invasive and capable of real-time online monitoring, can accurately capture the transient acoustic characteristics generated by pipeline leaks and valve failures, making it the mainstream NDT method for monitoring the health of offshore oil and gas pipelines. With the rise of the data-driven paradigm, intelligent structural health monitoring methods that combine acoustic emission sensing with deep learning have gradually emerged as a key technical approach for achieving early leak warning and condition monitoring of offshore pipelines [17,18,19]. Given that deep learning has been widely and successfully applied across various fields [20,21,22,23,24,25,26,27,28,29], its adoption for acoustic emission-based leak diagnosis in marine pipelines is well-justified and holds great promise.

In recent years, the integration of deep learning and acoustic emission (AE) technology has brought breakthrough progress in valve leak diagnosis for marine oil and gas pipelines [30,31,32,33,34,35]. Extensive exploratory studies have been conducted in this field. For instance, convolutional neural networks (CNNs) have been successfully applied to identify detailed waveform features of AE signals due to their powerful local feature extraction capabilities [36,37], while Transformer architectures have effectively captured global dependencies within long-sequence AE signals through the self-attention mechanism [38,39]. Furthermore, Saleem et al. proposed a hybrid CNN-LSTM deep learning framework for pipeline monitoring in smart city scenarios [40], and Kim et al. introduced a pipeline leak detection method based on continuous wavelet transform (CWT) and CNNs, which converts mass flow and pressure signals into time-frequency images [41]. However, existing deep learning methods face severe challenges in practical engineering applications, particularly in resource-constrained environments on offshore platforms. First, the cumbersome design of most models results in high computational costs, making them difficult to deploy on embedded devices [42]. Second, the environmental adaptability of these models is insufficient. Offshore platforms are persistently affected by strong background noise such as wave impacts and ocean current friction, along with multi-source interferences including equipment vibration and marine biological noise. Such noise components tend to mask the weak signals generated by pipeline leaks. Unfortunately, most existing models are designed for specific experimental conditions and are highly sensitive to variations in signal-to-noise ratio [43]. Third, large-scale deep learning models rely heavily on GPUs and large memory volumes, hindering their direct deployment on embedded monitoring systems or edge computing nodes of offshore platforms. The trade-off between theoretical performance and practical deployment efficiency, as well as the balance between diagnostic accuracy and computational overhead, has restricted the widespread implementation of intelligent monitoring for marine oil and gas pipelines [44,45,46,47].

To address the aforementioned limitations, lightweight design has become an important development trend in acoustic emission-based leak detection. Research on lightweight fault diagnosis models primarily focuses on two directions: model compression, which reduces the parameter count of existing models through pruning, quantization, and similar techniques, yet such methods inevitably sacrifice partial feature extraction capabilities and lead to accuracy degradation [48]; and lightweight module design, including depthwise separable convolutions and parameter-free attention mechanisms, which achieve lightweight performance at the structural level and represent a more promising technical path [49]. Ullah et al. [50] proposed a lightweight Vision Transformer (LViT-S) that reduces inference time while maintaining high accuracy by simplifying the self-attention mechanism, reducing embedding dimensions, and decreasing the number of Transformer layers. The lightweight neural network architecture introduced by Huang et al. [42] achieves high-representation capability of AE data while enabling leak monitoring of pipeline weld cracks by reducing sampling points, providing a feasible solution for offshore platform applications. Nevertheless, most existing lightweight studies are designed for general detection scenarios or other industrial equipment, and dedicated lightweight models for valve leakage detection in marine oil and gas pipelines remain scarce.

To tackle the above challenges, this paper proposes a Parameter-Free Star Attention Fusion Network (SAFNet) to achieve high-precision, lightweight, and end-to-end valve leak diagnosis for marine oil and gas pipelines, meeting the requirements of industrial process monitoring, rapid response, and safety management. SAFNet integrates four core complementary modules to form an end-to-end lightweight diagnostic framework. Specifically, this study first designs a Temporal Pyramid Encoder (TPE) to hierarchically extract deep features from raw valve leak AE signals. The TPE employs a five-level one-dimensional convolutional stack to capture the transient pulses and time-frequency distribution characteristics of leak AE signals in marine environments, laying a foundation for subsequent leak feature learning. Subsequently, a Parameter-Free Lightweight Star-shaped Attention (PLSA) module is constructed, whose core component is the built-in Dual Bilinear Star Mapping (DBSM) unit. This unit realizes parameter-free nonlinear transformation via dual linear mapping and element-wise multiplication, which specializes in mining feature differences between leak AE signals and marine background noise, thereby effectively distinguishing weak leak signals submerged in noise. Finally, a Multi-Scale Gated Attention Fusion (MS-GAF) module is introduced to adaptively aggregate multi-scale features output by multi-level PLSA modules and dynamically adjust the contribution weights of different hierarchical features, enhancing the representation of weak leak features that are easily masked in marine environments. Furthermore, an Energy-Driven Feature Refiner (EDFR) is embedded in both PLSA and MS-GAF modules, which adaptively assigns feature attention weights based on a neuron energy function. Without introducing additional learnable parameters, the EDFR suppresses redundant features caused by marine noise and highlights discriminative features related to leakage faults. The main contributions of this paper are summarized as follows:

1. This study proposes the Parameter-Free Star Attention Fusion Network (SAFNet). Its core Parameter-Free Lightweight Star-shaped Attention (PLSA) module directly generates attention weights via an analytical energy function and maps inputs to a high-dimensional nonlinear space, effectively decoupling complex nonlinear relationships in data. This enables lightweight, high-precision, and real-time identification of valve leak AE signals, achieving an excellent balance among accuracy, inference speed, and robustness.
2. To verify the engineering applicability of SAFNet in real marine environments, this study constructs an AE dataset that combines laboratory leak signals and on-site marine noise. Pure leak signals are collected through controlled pipeline valve leak experiments, while in situ marine noise is acquired from the Liwan 3-1 platform in the South China Sea. Linear reconstruction is performed to generate realistic marine pipeline leak AE data. This dataset compensates for the lack of real marine interference in existing public datasets and provides high-quality engineering support for the training and validation of SAFNet.
3. This study designs a comprehensive experimental evaluation covering hyperparameter selection, performance metrics, robustness, and computational complexity, which systematically validates the effectiveness and superiority of SAFNet. Experimental results demonstrate that SAFNet achieves only 0.73 M parameters and 0.46 M floating-point operations, with a single-sample inference time of 313.81 μs. The diagnostic accuracy remains stable at 96.5–98.5% under 2–5 MPa working conditions, and exceeds 88% even in an extreme 0 dB noise environment, outperforming all compared models. This provides a practical technical solution for edge intelligent monitoring in marine engineering applications.

## 2. Methodology

This section elaborates on the proposed SAFNet fault diagnosis method, which mainly consists of a Temporal Pyramid Encoder (TPE), a multi-stage Parameter-free Lightweight Star-shaped Attention (PLSA) module, a Multi-Scale Gated Attention Fusion (MS-GAF) module, an Energy-Driven Feature Refiner (EDFR), and a leak detection classifier. The model takes one-dimensional acoustic emission (AE) data as input. After deep feature extraction by the TPE encoder, the signal is fed into three cascaded PLSA modules to generate multi-scale features. These multi-scale features are then adaptively fused by the MS-GAF module. Finally, the fault category is output through the classifier.

### 2.1. Symbol Definitions

To ensure the rigor of mathematical derivations and consistency in notation usage, all variables, parameters, and feature quantities are explicitly defined in this section. Each symbol is explained in detail upon its first appearance, and a unified notation is strictly maintained throughout subsequent chapters. For clarity and consistency, the key symbols used in this section are summarized in Table 1.

### 2.2. Temporal Pyramid Encoder (TPE)

Acoustic Emission (AE) signals contain broadband information ranging from low-frequency envelopes to high-frequency transient impulses, with fault features often appearing at different temporal scales. These typically manifest as sustained high-frequency oscillations for small leaks and sudden transient impacts for large leaks. Therefore, we first design a hierarchical multi-scale temporal feature extraction module to construct a feature pyramid from raw AE signals. This provides the subsequent star-shaped attention modules with base features rich in time-frequency information, adapting to the multi-scale characteristics of leak signals that combine both transient pulses and steady-state oscillations.

The TPE is composed of 5 stacked 1D convolutional layers. The core mathematical expression for the convolution operation at each level is given by:

(1) $$F_{\mathrm{conv}}\left(b,c,t\right)=\left(F_{\mathrm{in}}*k\right)\left(b,c,t\right)+b_{0}$$

where $F_{\mathrm{conv}}\left(b,c,t\right)$ is the feature value of the output feature map at batch *b*, channel *c*, and time step *t*, $b_{0}$ is the fixed bias term, and ∗(·) denotes the 1D convolution operation, which is defined as:

(2) $$\left(F*k\right)\left(t\right)=\sum_{\tau =0}^{k-1}F\left(t-\tau \right)k\left(\tau \right)$$

The convolution kernel size is set to k=3, stride s=2, and padding p=1, to ensure both the completeness of feature extraction and downsampling efficiency. After each convolution operation, the length of the temporal dimension is progressively downsampled according to the following formula, while the number of channels is doubled at each stage:

(3) $$L^{\prime }=\frac{L+2p-k}{s}+1$$

where *L* is the temporal length of the input feature, and $L^{\prime }$ is the temporal length of the output feature. After five convolution layers, the number of channels is adjusted, and the final output base feature map is $F_{\mathrm{TPE}}\in R^{B\times C_{5}\times L_{5}}$. This reduces the computational load for subsequent modules while preserving key time-frequency information.

### 2.3. Parameter-Free Lightweight Star-Shaped Attention (PLSA) Module

In this study, the PLSA module is designed as the core feature extraction unit of the network. Following a modular design philosophy, it consists of four synergistic components: depthwise separable convolution, Dual Bilinear Star Mapping (DBSM), residual connections, and an Energy-Driven Feature Refiner (EDFR). Multi-scale feature enhancement is achieved by stacking $N_{\mathrm{PLSA}}$ such modules, where $N_{\mathrm{PLSA}}$ is a hyperparameter to be optimized. The optimal number of stacked modules is determined via hyperparameter sensitivity analysis to adapt to feature extraction requirements under different working conditions.

DBSM is the core functional component of PLSA. Inspired by star topology structures, it captures the nonlinear coupling relationships between the core leak signal and marine noise through interactions between central and peripheral features, addressing the challenge of distinguishing weak features under strong background noise. For an input feature map $F_{\mathrm{dwconv}}\in R^{B\times C\times L}$, it is split along the channel dimension into one central feature channel and C−1 peripheral feature channels to construct the star topology:

(4) $$F_{c}\left(b,:,:\right)=F_{\mathrm{dwconv}}\left(b,0,:\right)$$

(5) $$F_{p,i}\left(b,:,:\right)=F_{\mathrm{dwconv}}\left(b,i,:\right)\;\left(i=1,2,\ldots ,C-1\right)$$

where $F_{c}$ is the star-center feature corresponding to the transient core leak signal, and $F_{p,i}$ are the star-edge features corresponding to environmental noise or signal redundancy. The star topology embodies the global interaction between the central feature and all peripheral features, rather than only local feature associations.

Subsequently, nonlinear interactions between the central feature and each peripheral feature are realized through linear mappings with fixed weight matrices and element-wise multiplication, without introducing additional learnable parameters. The mathematical formulation is as follows:

(6) $$F_{\mathrm{int},i}\left(b,:,:\right)=\left(F_{c}\cdot W_{c}\right)⊙\left(F_{p,i}\cdot W_{p}\right)$$

where $W_{c},W_{p}\in R^{L\times L}$ are fixed weight matrices. Through this bilinear interaction between the central and peripheral features, the module implicitly models their nonlinear correlations, enhancing feature representation without expanding the network dimension, thereby balancing lightweight design and performance. Then, the interaction results between all peripheral features and the central feature are summed to aggregate the global information of the star topology, outputting the final mapped feature:

(7) $$F_{\mathrm{DBSM}}\left(b,:,:\right)=\sum_{i=1}^{C-1}F_{\mathrm{int},i}\left(b,:,:\right)$$

This aggregation process preserves the central position of the leak feature while integrating the correlation information of the peripheral features, achieving effective decoupling between the leak signal and noise.

The EDFR is the feature optimization component of PLSA. Based on a neuron energy function, it adaptively suppresses noise redundancy in the marine environment and enhances leak-related discriminative features without introducing additional learnable parameters, ensuring the lightweight nature of the module. First, the mean and variance of each channel of the feature map $F_{\mathrm{res}}\in R^{B\times C\times L}$ after the residual connection are calculated to quantify the feature distribution characteristics, providing statistical support for subsequent attention weight generation.

We first define the mean $\mu_{c}$ of the *c*-th channel as the average of all feature values across all batches and time steps within that channel. Its mathematical expression is given by:

(8) $$\mu_{c}=\frac{1}{B\times L}\sum_{b=1}^{B}\sum_{t=1}^{L}F_{\mathrm{res}}\left(b,c,t\right)$$

Furthermore, the variance $\sigma_{c}^{2}$ of the *c*-th channel is defined as the mean squared deviation of the feature values from their mean, which measures the dispersion of the feature distribution. Its mathematical expression is given by:

(9) $$\sigma_{c}^{2}=\frac{1}{B\times L}\sum_{b=1}^{B}\sum_{t=1}^{L}{\left(F_{\mathrm{res}}\left(b,c,t\right)-\mu_{c}\right)}^{2}$$

Based on the channel mean and variance obtained from the above statistics, we define an energy importance score $S_{c}$ to measure how far feature values deviate from the channel mean. A larger deviation indicates that the channel features contain more leak-related valid information. Its mathematical expression is given by:

(10) $$S_{c}=\frac{|\mu_{c}|}{\sigma_{c}+\epsilon }$$

where ϵ is a regularization constant to avoid division by zero. To convert this score into attention weights that can be directly used for feature weighting, $S_{c}$ is then mapped to the interval [0,1] using the sigmoid function, yielding the attention weight $W_{\mathrm{att}}\left(c\right)$ for the *c*-th channel. This enables adaptive enhancement of effective features and suppression of noisy features. The mathematical expression is as follows:

(11) $$W_{\mathrm{att}}\left(c\right)=\frac{1}{1+e^{-S_{c}}}$$

Finally, the generated attention weights are multiplied element-wise with the input feature map $F_{\mathrm{res}}$ to weight-adjust the features of each channel, yielding the refined feature map $F_{\mathrm{EDFR}}$. Its mathematical expression is given by:

(12) $$F_{\mathrm{EDFR}}\left(b,c,t\right)=F_{\mathrm{res}}\left(b,c,t\right)⊙W_{\mathrm{att}}\left(c\right)$$

To reduce computational complexity, PLSA uses depthwise separable convolution instead of standard convolution to perform lightweight feature adjustment. The mathematical expression is given by:

(13) $$F_{\mathrm{dwconv}}\left(b,c,t\right)=\sum_{\tau =0}^{k-1}F_{\mathrm{in}}\left(b,c,t-\tau \right)k_{c}\left(\tau \right)$$

where $K_{c}$ is the convolution kernel specific to the *c*-th channel, which performs local feature extraction only within the individual channel, avoiding redundant computations across channels.

Subsequently, to mitigate the vanishing gradient problem in deep networks and preserve the original feature information, PLSA introduces a residual connection, directly adding the DBSM output to the module input feature:

(14) $$F_{\mathrm{res}}\left(b,:,:\right)=F_{\mathrm{DBSM}}\left(b,:,:\right)+F_{\mathrm{in}}\left(b,:,:\right)$$

The residual connection ensures that the star-mapped features do not lose basic information, while enhancing gradient propagation and improving the training stability of the model.

### 2.4. Multi-Scale Gated Attention Fusion (MS-GAF) Unit

Since the signal features of different leak levels are distributed across different temporal scales, we design the MS-GAF unit to adaptively aggregate the multi-scale features output by $N_{\mathrm{PLSA}}$ PLSA modules through a gating mechanism. It dynamically adjusts the contribution weights of each scale to address the pain point where weak features are hidden in middle-level semantics.

First, the number of channels of all PLSA output feature maps is aligned through linear transformations to obtain $F_{P_{1}}^{\prime },F_{P_{2}}^{\prime },\ldots ,F_{P_{N}}^{\prime }$, ensuring consistent dimensions for fusion.

Then, the global information of each scale feature is extracted via global average pooling to compute the adaptive gating weights:

(15) $$G_{i}=\frac{1}{B\times L}\sum_{b=1}^{B}\sum_{t=1}^{L}F_{P_{i}}^{\prime }\left(b,:,:\right)\;\left(i=1,2,\ldots ,N\right)$$

where $G_{i}$ denotes the global feature vector of the *i*-th PLSA output feature after global average pooling. An adaptive gating weight is then obtained through a normalization operation, ensuring that the sum of the weights of all scales is 1, enabling interpretable assignment of importance:

(16) $$\alpha_{i}=\frac{G_{i}}{\sum_{j=1}^{N_{\mathrm{PLSA}}}G_{j}}\;\left(i=1,2,\ldots ,N\right)$$

where $\sum_{i=1}^{N}\alpha_{i}=1$, and the magnitude of the weights reflects the discriminative importance for the final leak identification task.

After obtaining the adaptive gating weights for features at each scale, we implement adaptive aggregation of multi-scale features through a weighted summation operation. This highlights the feature contributions of scales with high importance while suppressing redundant information from scales with low importance:

(17) $$F_{\mathrm{fusion}}\left(b,:,:\right)=\sum_{i=1}^{N_{\mathrm{PLSA}}}\alpha_{i}\cdot F_{P_{i}}^{\prime }\left(b,:,:\right)$$

where $F_{\mathrm{fusion}}$ is the intermediate feature map after multi-scale feature aggregation. To further suppress redundant features caused by marine environmental noise and enhance leak-related discriminative features, the aggregated feature map is fed into the EDFR module for refinement. Finally, the global feature $F_{\mathrm{global}}\in R^{B\times C_{\mathrm{final}}\times L_{\mathrm{final}}}$ is obtained for subsequent fault diagnosis.

### 2.5. Leak Detection Classifier

The global feature $F_{\mathrm{global}}$ retains multi-scale temporal discriminative information. However, the 3D tensor structure cannot be directly fed into the classifier for fault type identification. Therefore, we first need to compress the temporal dimension through global average pooling to aggregate variable-length temporal features into fixed-length channel-wise features. Then, through a linear transformation and probability normalization, the final fault prediction probabilities are output.

First, global average pooling is performed on the temporal dimension of the global feature to compress the 3D feature into a 2D feature, while preserving discriminative information between channels to eliminate the impact of temporal length differences on classification:

(18) $$F_{\mathrm{pool}}\left(b,c\right)=\frac{1}{L_{\mathrm{final}}}\sum_{t=1}^{L_{\mathrm{final}}}F_{\mathrm{global}}\left(b,c,t\right)$$

where $F_{\mathrm{pool}}\left(b,c\right)$ denotes the pooled feature of the *b*-th sample and the *c*-th channel. By taking the average over the temporal dimension *t*, we aggregate temporal information into channel-wise statistics, obtaining a fixed-dimensional feature $F_{\mathrm{pool}}\in R^{B\times C_{\mathrm{final}}}$. This provides a stable input for subsequent linear transformations. As the core evidence for the classifier, this feature condenses the discriminative contribution of each channel to leak localization.

The pooled 2D decision features are further mapped to a fault class probability distribution: the channel dimension is mapped to the number of fault classes *N* via a linear layer, and then the linear output is normalized to probability values in the range [0,1] through the Softmax function, yielding the fault prediction probability for each sample:

(19) $$P\left(b,n\right)=\frac{\exp\left(F_{\mathrm{pool}}\left(b,:\right)W_{\mathrm{fc}}\left(:,n\right)+b_{\mathrm{fc}}\left(n\right)\right)}{\sum_{n^{\prime }=1}^{N}\exp\left(F_{\mathrm{pool}}\left(b,:\right)W_{\mathrm{fc}}\left(:,n^{\prime }\right)+b_{\mathrm{fc}}\left(n^{\prime }\right)\right)}$$

where $W_{\mathrm{fc}}\in R^{C_{\mathrm{final}}\times N}$ and $b_{\mathrm{fc}}\in R^{N}$ are the learnable parameters of the linear layer. The cross-entropy loss function is used for optimization during training. P(b,n) denotes the probability that the *b*-th sample belongs to the *n*-th leak valve, satisfying $\sum_{n=1}^{N}P\left(b,n\right)=1$.

The core objective of the classifier is to output a specific leak valve ID. Therefore, the final decision is made based on the confidence distribution by selecting the class with the highest confidence as the localization result. The final localization decision result $n^{*}$ is given by:

(20) $$n^{*}=\arg\max_{n\in \{1,2,\ldots ,N\}}P\left(b,n\right)$$

### 2.6. Overall Architecture

To simplify the complex and diverse marine oil and gas pipeline leak detection problem, we formulate the leak valve detection task as a multi-class classification problem. For a raw acoustic emission input signal of length *L*, shallow multi-scale features are first extracted by the Temporal Pyramid Encoder. Subsequently, the features pass through *n* stacked Parameter-free Lightweight Star-shaped Attention (PLSA) modules to gradually refine deep semantic features. The Multi-Scale Gated Attention Fusion (MS-GAF) module then adaptively fuses the information from these *n* scales to generate a global feature vector. Finally, the classifier outputs the probability of each fault class.

**Figure 1:**
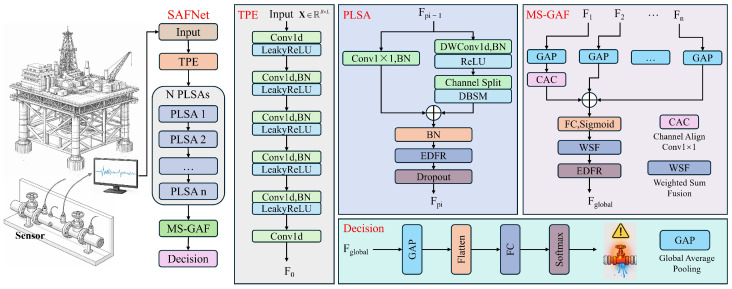
SAFNet Architecture Diagram.

This design avoids the computational overhead of sub-networks in traditional attention mechanisms, while effectively capturing and decoupling the feature information of leak locations in marine oil and gas pipelines. The complete architecture of the proposed SAFNet is shown in Figure 1.

## 3. Experimental Setup and Dataset

This section systematically and comprehensively describes the methodological framework for investigating internal valve leakage in marine oil and gas pipelines. The framework covers the experimental configuration, data acquisition, data fusion methods, dataset preparation procedures, computational infrastructure, and performance evaluation metrics.

### 3.1. Data Acquisition

This study adopts a two-stage experimental approach to characterize the internal leakage behavior of valves in marine oil and gas pipelines. In the first stage, controlled laboratory experiments are conducted to collect pure leakage signals. In the second stage, on-site noise signals are acquired from the offshore platform. By linearly fusing these two types of signals, realistic marine pipeline valve leakage scenarios can be accurately simulated.

**Figure 2:**
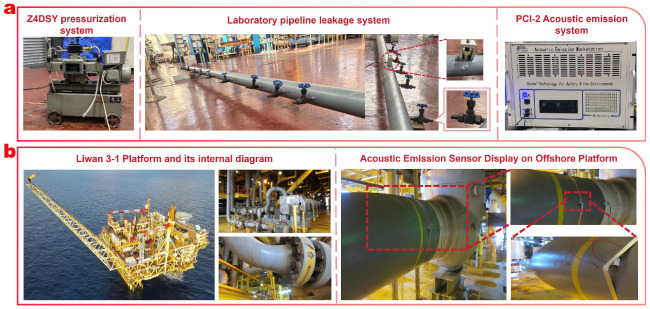
Experimental setup. (**a**) Schematic of the laboratory setup for simulating pipeline leakage. (**b**) Layout for noise signal acquisition on the offshore platform.

The specific scheme for the laboratory pipeline leakage signal acquisition phase is as follows: The experimental pipeline uses seamless steel pipes conforming to the GB/T 8163-2018 [51] standard, with a length of 6.2 m, a nominal diameter of 105 mm, and a wall thickness of 5 mm, to simulate the high-pressure fluid transportation conditions of marine oil and gas pipelines. Ten high-pressure needle control valves are evenly installed along the pipeline to simulate internal leakage conditions occurring at different valve positions. With the help of the Z4DSY pressurization system (manufactured in China), the internal pressure of the pipeline is precisely adjusted to four gradients: 2 MPa, 3 MPa, 4 MPa, and 5 MPa, to quantify the influence of different pressure conditions on the internal leakage characteristics of the valves. The configuration of the experimental system in this stage is shown in Figure 2a.

The acoustic emission signal acquisition system built in this study mainly consists of a PCI-2 data acquisition device, R15 acoustic emission sensors, and preamplifiers (Physical Acoustics, Mistras Group, Princeton Junction, NJ, USA). The operating frequency range of the PCI-2 device is 1 kHz to 3 MHz; the center frequency of the R15 sensor is set to 150 kHz; and the gain of the preamplifier is adjusted to 40 dB to achieve effective signal amplification. The sampling frequency of the system is configured to 1 MHz, and the threshold voltage is set to 0.1 V to ensure the optimal signal-to-noise ratio during signal acquisition. To suppress the interference of environmental noise on the effective signal, the collected raw acoustic emission signals are sequentially processed with high-pass and low-pass filtering. The sensor is deployed 10 cm from the downstream boundary, using industrial white petroleum grease (manufactured in China) as a couplant to ensure close contact with the test surface, which guarantees efficient transmission of acoustic emission signals between media. The spatial positions of each valve relative to the sensor are detailed in Table 2. With the above system configuration, high-precision capture of the acoustic emission signals from pipeline valve leaks can be achieved while minimizing background noise interference. The sensor deployed downstream enables full-coverage signal acquisition for leakage events from all valves.

For the noise signal acquisition stage, the Liwan 3-1 offshore platform in the South China Sea was selected as the field test site. The platform is an eight-legged jacket steel structure anchored to the seabed at a water depth of 191 m through 16 piles. It has three main decks at elevations of 19 m, 29 m, and 41 m, with a topside weight of 32,000 t. Combined with field surveys and analysis of the platform’s actual operating conditions, significant vibration and stress concentrations were identified at the natural gas export riser on the 19 m deck, which are prone to structural fatigue damage. Therefore, the transverse weld area of this riser was designated as the key monitoring location. Subsequently, R15 acoustic emission sensors with the same specifications and parameters as those used in the laboratory tests were deployed evenly at preset positions, as shown in Figure 2b. Sensor parameters were strictly set according to the laboratory standards. Based on this setup, a long-term automated data acquisition program was initiated, and a large dataset of on-site noise signals was collected from 16 to 27 June 2024.

### 3.2. Dataset Description and Partitioning

The acoustic emission (AE) dataset used in this study contains samples evenly distributed across 10 different valve positions for each operating condition. This balanced sampling design comprehensively covers the leakage-related signal variations under different valve configurations, laying a solid data foundation for the stable training and evaluation of SAFNet. To eliminate data distribution bias and ensure the reliability of SAFNet performance evaluation, a stratified sampling strategy was adopted, dividing the dataset into training and test sets at a fixed ratio of 8:2. The valve position was chosen as the stratification criterion, as it is the core factor determining the time-domain and frequency-domain characteristics of pipeline leakage-related AE signals.The partitioning details of the acoustic emission (AE) Dataset are summarized in Table 3.

### 3.3. Evaluation Metrics and Experimental Settings

Two main evaluation metrics were used in this study: accuracy and F1-score. These metrics are quantified using the following mathematical expressions [52]:

(21) $$\mathrm{Accuracy}=\frac{TP+TN}{TP+TN+FP+FN}$$

(22) $$F1\text{-}\mathrm{score}=2\times \frac{\mathrm{Precision}\times \mathrm{Recall}}{\mathrm{Precision}+\mathrm{Recall}}$$

*TP* denotes true positives, TN denotes true negatives, FP denotes false positives, and FN denotes false negatives. The experimental evaluations in this study were conducted on a computing platform equipped with an Intel i7-10750H processor, an NVIDIA Laptop GTX 1650 Ti graphics card, and 16 GB of system memory.

## 4. Results and Discussion

### 4.1. Signal Feature Analysis

**Figure 3:**
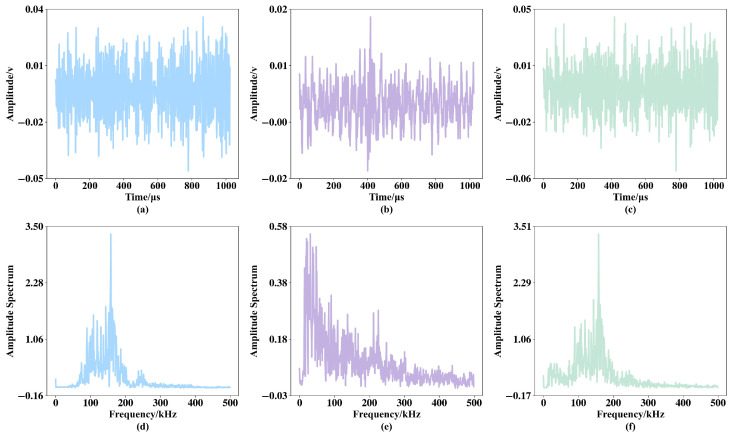
Time-frequency domain comparison of acoustic emission signals: (**a**) Time-domain waveform of the laboratory valve leakage signal, (**b**) Time-domain waveform of the offshore platform noise signal, (**c**) Time-domain waveform of the fused signal, (**d**) Spectrum of the laboratory leakage signal, (**e**) Spectrum of the offshore platform noise signal, (**f**) Spectrum of the fused signal.

To verify that the dataset constructed via the proposed signal fusion strategy can effectively simulate real-world offshore conditions, this subsection investigates the characteristics of the collected leak and noise signals. It details the signal fusion process used to simulate a realistic marine environment and analyzes the properties of multi-valve leak signals. Representative signals from laboratory leak measurements and offshore platform noise recordings were selected for comprehensive characterization. As shown in Figure 3, comparisons in both the time and frequency domains reveal distinct patterns.

In the time domain, Figure 3a shows the laboratory valve leakage acoustic emission signal, which exhibits typical sparse high-amplitude pulse characteristics with an amplitude range of −0.04 to 0.04 V. Short pulses correspond to transient fluid impacts caused by leakage, while low-amplitude continuous oscillations reflect turbulent steady states. Figure 3b shows the offshore platform on-site noise signal, whose time-domain waveform presents random wide-amplitude fluctuations with an amplitude range of −0.02 to 0.02 V, showing no obvious periodic or pulsed structure and reflecting the superposition effect of multi-source interference. Figure 3c shows the fused signal, whose amplitude range extends to −0.04 to 0.05 V. It retains both the leakage pulse characteristics and the random fluctuations of on-site noise, significantly increasing signal complexity and accurately simulating the masking effect of ocean noise on leakage signals. In the frequency domain, Figure 3d shows the spectrum of the laboratory leakage signal, whose energy is highly concentrated with clear characteristic peaks corresponding to the core frequency band of the leak. Figure 3e shows the spectrum of the offshore platform noise, which presents a broadband low-energy distribution with no obvious concentrated frequency band, reflecting the wide-spectrum interference characteristics. Figure 3f shows the spectrum of the fused signal. Although the leakage characteristic peaks are retained, the overall energy is elevated by noise, leading to a high degree of overlap between the leakage frequency band and the noise frequency band. The identifiability of the leakage features is significantly reduced, greatly increasing the difficulty of leak detection in complex environments. This fusion strategy constructs a comprehensive dataset that retains the essential acoustic characteristics of valve leakage while incorporating real ocean environmental interference, providing a high-quality data foundation that closely matches engineering reality for subsequent training and validation.

**Figure 4:**
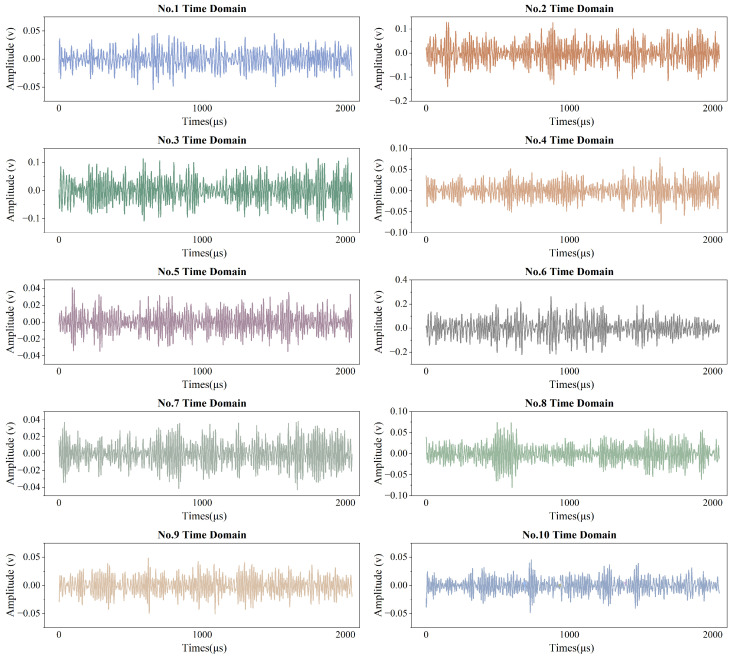
Time-domain waveforms of acoustic emission signals for valve leakage at positions 1 to 10.

Further research focuses on the acoustic emission signal characteristics of the 10 high-pressure needle valves under leakage conditions. As shown in Figure 4, time-domain waveform analysis reveals unique patterns among the leakage signals from different valves. The time-domain measurement data exhibit typical non-stationary behavior. During the 2000 μs sampling period, the valve signals continuously present random oscillations accompanied by significant amplitude variations. Although the signal morphologies at different valve positions are similar, subtle differences exist in local amplitude intensity and oscillation frequency. These subtle variations highlight the limitations of relying solely on time-domain features for precise leak localization. For example, the No. 1 valve exhibits sparse pulse density and minimal peak-to-peak variation, reflecting early micro-leakage or low-flow operating conditions. The No. 2 valve is characterized by moderate amplitude fluctuations and dense or phase-concentrated high-frequency pulses, corresponding to seal wear, turbulence, or intermittent changes in valve opening. The No. 6 valve features an extremely large amplitude range with sudden high-intensity transient spikes, indicating potential severe structural faults or transient impact events. The diverse leakage characteristics of different valves provide a foundation for developing anti-noise acoustic emission detection algorithms suitable for marine environments, facilitating early identification of high-risk leakage conditions and mitigating threats to offshore process safety and marine ecological integrity.

**Figure 5:**
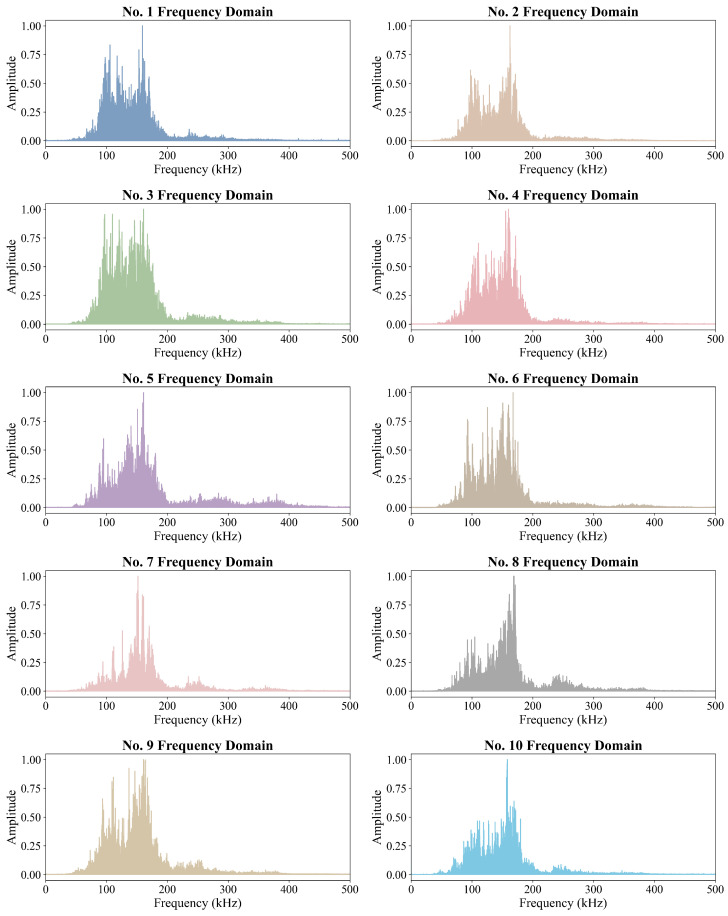
Frequency spectra of acoustic emission signals for leakage at valve positions 1 to 10.

As illustrated in Figure 5, the frequency-domain analysis of valve leakage signals reveals distinct spectral characteristics. The spectral energy is primarily concentrated within the range of 0–500 kHz, with significant energy accumulation observed in the 100–200 kHz frequency band. Furthermore, substantial overlap exists among the dominant frequency components of all valve leakage signals. The signals consistently exhibit the maximum amplitude at approximately 150 kHz, followed by a gradual energy decay at higher frequencies. These highly similar spectral characteristics indicate that traditional frequency-domain analysis methods suffer from inherent limitations in effectively distinguishing leakage signals generated by different valves.

### 4.2. Hyperparameter Sensitivity Analysis

**Figure 6:**
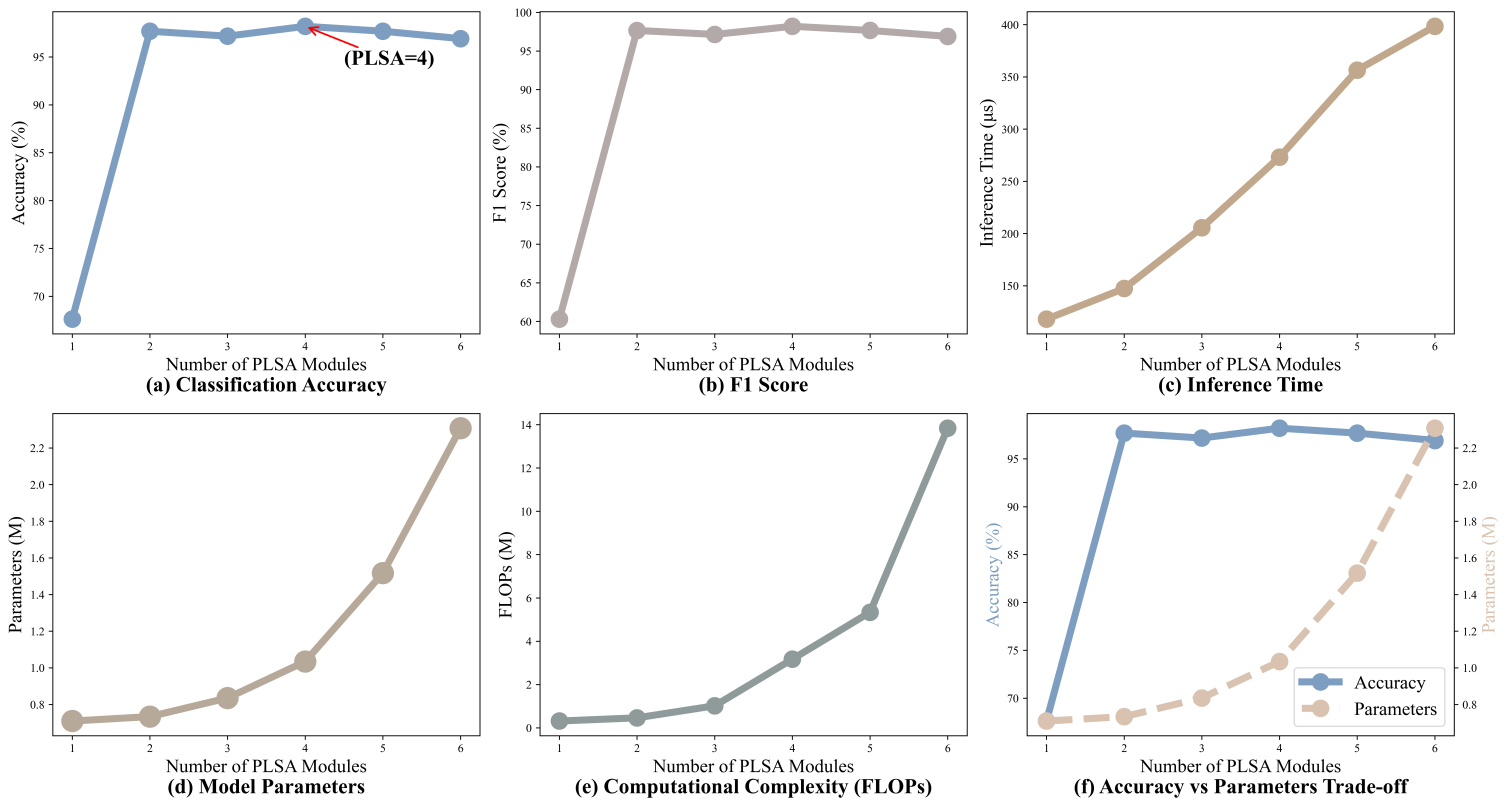
Sensitivity analysis of the number of PLSA modules on the leakage detection performance of SAFNet for marine oil and gas pipelines: (**a**) Classification accuracy. (**b**) Weighted F1-score. (**c**) Single-sample inference time. (**d**) Number of parameters. (**e**) Computational complexity (FLOPs, millions). (**f**) Trade-off curve between classification accuracy and number of parameters.

To verify the rationality of the SAFNet network structure configuration and its robustness under hyperparameter perturbations, this section systematically evaluates the effects of the number of PLSA modules, learning rate, and batch size on model performance and efficiency. As shown in Figure 6a, the classification accuracy exhibits the typical diminishing marginal benefit characteristic: it rises rapidly and then saturates as the number of PLSA modules increases. When the number of modules increases from 1 to 2, the accuracy jumps from 67.8% to 98.2%, indicating that the initially stacked PLSA modules can effectively enhance the ability to extract weak leakage features from acoustic emission signals. The accuracy peaks at 4 modules and remains stable above 97.5% when further increased to 6, with the performance gain gradually converging.

The weighted F1-score in Figure 6b shows a highly consistent trend with accuracy, stabilizing in the range of 96% to 98% when the number of PLSA modules is greater than 2. This verifies the robust balance between precision and recall achieved by SAFNet in the leak detection task. Meanwhile, in terms of computational efficiency, increasing the number of modules linearly increases the inference latency. Figure 6c shows that the latency grows linearly, from 120 μs with 1 PLSA module to 398 μs with 6 PLSA modules.

Figure 6d,e show that the number of parameters of SAFNet increases from 0.72 M to 2.32 M, while the computational complexity surges from 0.3 M to 14.1 M, an increase of over 46 times. This indicates that too many modules lead to a significant rise in computational load. The accuracy vs. parameter count trade-off curve in Figure 6f further clarifies that 2 modules represent the optimal engineering configuration. This scheme achieves extremely high classification accuracy while maintaining a lightweight parameter count and controllable inference latency, striking the best balance between discriminative capability and computational cost. It enables both high-precision diagnosis and efficient real-time inference in process safety applications for marine oil and gas pipeline leak detection.

**Figure 7:**
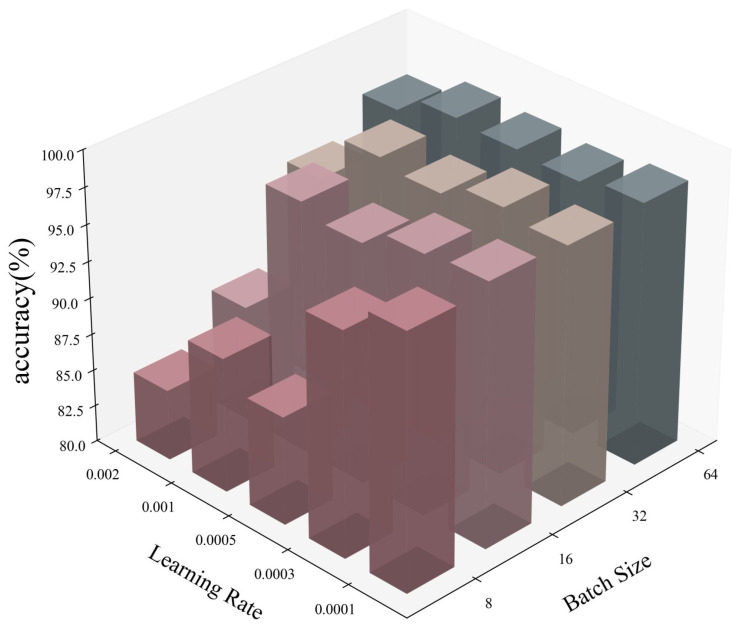
Classification accuracy of the model under different combinations of learning rate and batch size. Light reddish bars, light tan bars, and dark gray bars represent small, medium and large learning rate groups, respectively.

Figure 7 intuitively demonstrates the effects of two core training hyperparameters, namely learning rate and batch size, on the classification accuracy of SAFNet for the marine oil and gas pipeline leak detection task. SAFNet achieves the highest classification accuracy when the batch size is 32 and the learning rate is 0.0003, representing the optimal hyperparameter combination for this task. Overall, SAFNet maintains an accuracy of over 90% under the vast majority of hyperparameter configurations. Obvious performance degradation only occurs under extreme conditions, such as an excessively large learning rate or an extremely small batch size. This fully proves the low sensitivity of SAFNet to training hyperparameters.

**Figure 8:**
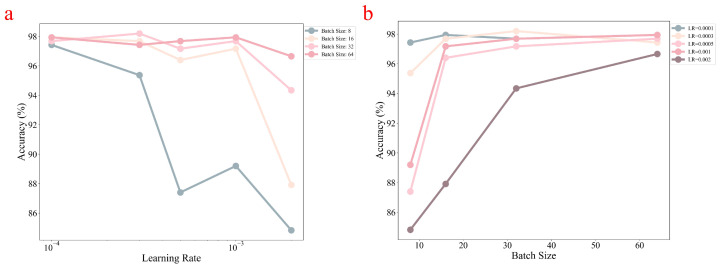
Analysis of the influence of learning rate and batch size on model classification accuracy. (**a**) Classification accuracy curves of different batch sizes under varying learning rates; (**b**) Classification accuracy curves of different learning rates under varying batch sizes.

To further quantify the effects of learning rate and batch size on SAFNet performance and reveal the patterns of how different hyperparameter combinations influence SAFNet classification accuracy, Figure 8a,b conduct hyperparameter sensitivity validation from two dimensions. The results show that batch size plays an important role in the robustness of SAFNet. When the batch size is greater than or equal to 16, SAFNet maintains a high accuracy of over 97% across the learning rate range from 0.0001 to 0.001, with only a slight decrease observed when the learning rate exceeds 0.001. In contrast, when the batch size is 8, SAFNet is extremely sensitive to changes in learning rate: accuracy drops rapidly once the learning rate exceeds 0.0005, falling below 85%.

From the perspective of batch size, under the condition that the learning rate is greater than or equal to 0.001, SAFNet accuracy shows a stepwise increase as the batch size increases. When the batch size increases from 8 to 32, the accuracy improves by nearly 10%. When the learning rate is less than or equal to 0.0005, SAFNet performance quickly saturates as the batch size increases, reaching its peak at a batch size of 32 with no further significant gains when increased to 64.

These results are fully consistent with the conclusions from the 3D bar chart, jointly confirming that a batch size of 32 and a learning rate of 0.0003 constitute the optimal training configuration for SAFNet. At the same time, they fully verify the strong robustness of SAFNet to learning rate variations under medium and large batch settings.The robustness of the proposed SAFNet to variations in hyperparameters such as the number of PLSA modules, batch size, and learning rate fully demonstrates its low sensitivity to training hyperparameter configurations. This reduces the cost of hyperparameter tuning and improves the operational reliability of the leak monitoring system in practical deployments.

### 4.3. Comparison with Learnable Attention Mechanisms

To isolate the contribution of the parameter-free design in PLSA and to investigate whether its performance gains originate from the attention formulation itself or merely from architectural simplification, we conducted a controlled experiment by replacing PLSA in SAFNet with three representative lightweight learnable attention modules: Squeeze-and-Excitation (SE) [53], Efficient Channel Attention (ECA) [54], and Convolutional Block Attention Module (CBAM) [55]. For a fair comparison, we adjusted the reduction ratios and kernel sizes of each attention module to ensure that the total parameter count of all variants remained within the range of 0.72–0.74 M, closely matching the original SAFNet. All models were trained under identical configurations, including learning rate, batch size, number of epochs, and data splits, with three independent runs for each variant to ensure statistical reliability.

As summarized in Table 4, several noteworthy observations can be drawn from the ablation results. First, ECA attains the maximum classification accuracy of 98.46% and the maximum F1-score of 98.45% across all tested attention mechanisms. This lightweight learnable attention module surpasses the original PLSA by only 0.26 percentage points on classification precision, which verifies that ECA acts as a competitive baseline for channel feature extraction tasks based on acoustic emission leakage signals. Second, the original PLSA delivers the shortest single-sample inference latency at a value of 2.604 ms among all model variants. When compared with the ECA variant, PLSA cuts inference latency by 13.0 percent; the fixed inference latency of ECA stands at 2.992 ms, while the corresponding latency of PLSA is 2.604 ms. In exchange for this prominent speed gain, PLSA only loses 0.26 percentage points of classification accuracy, forming a precision-speed trade-off that perfectly fits the demand of real-time edge monitoring hardware. The SE variant and CBAM variant produce classification performance comparable to or marginally inferior to that of the original PLSA, but they introduce significantly heavier computational burdens. The single-sample inference latency of the SE variant reaches 2.763 ms, and the single-sample inference latency of the CBAM variant reaches 3.703 ms. Such prolonged inference time makes these two attention modules less applicable to offshore pipeline monitoring nodes with tight resource limits.Third, the fact that a learnable attention mechanism (ECA) can marginally surpass PLSA in accuracy does not diminish the value of our parameter-free design. Rather, it highlights a fundamental design choice: for applications where latency is critical and memory bandwidth is limited, the parameter-free formulation offers a superior solution. On offshore platform edge devices, where continuous 24/7 monitoring is required under strict power budgets, the 13% latency reduction and zero parameter overhead of PLSA translate directly into lower energy consumption and higher system throughput, without materially compromising diagnostic accuracy. It is also worth noting that CBAM achieves exactly the same accuracy as PLSA while requiring 0.0103 M additional parameters and a 42.2% longer inference time, further reinforcing the computational efficiency advantage of our star-shaped attention design. SE, on the other hand, underperforms both PLSA and ECA across all metrics, suggesting that the two-layer fully-connected structure in SE introduces redundant capacity without commensurate performance gains.

In summary, while PLSA does not achieve the absolute highest accuracy among all compared attention mechanisms, it delivers the most favorable accuracy-efficiency trade-off, which is the central design objective for practical edge deployment scenarios. The 0.26% accuracy gap with ECA is well justified by the 13% inference speedup and the complete elimination of learnable parameters in the attention module.

### 4.4. Experimental Results and Model Comparison

This section evaluates in detail the performance of the proposed SAFNet in marine oil and gas pipeline valve leak localization. We conduct a comprehensive comparison of SAFNet with several representative baseline models, including CNN-LSTM [56], ECA-Net [57], MobileNet [58], ResNet18 [59], and ShuffleNetV2 [60]. To ensure reliable evaluation results, each model is tested five times independently, and the final results are averaged.

Table 5 presents the performance of various models for marine oil and gas pipeline valve leak localization. Overall, the proposed SAFNet outperforms all mainstream deep learning models across both diagnostic accuracy and computational efficiency metrics. In terms of classification performance, SAFNet achieves state-of-the-art results on both the average accuracy (ACC) and F1-score. Specifically, SAFNet yields an average ACC of 97.69% and an F1-score of 97.68%, matching the performance of the best competing model (ECA-Net) while significantly outperforming other approaches. Compared with the lowest-performing CNN-LSTM, SAFNet improves ACC by 21.85% and F1-score by 25.64%, demonstrating its superior capability to identify leakage patterns. Regarding computational efficiency, SAFNet exhibits clear advantages in both training and inference speed. The training time of SAFNet is only 72.69 s, which is approximately 56.9% faster than ECA-Net and 54.3% faster than ResNet18. While its inference time is not the fastest, it remains competitive and is significantly lower than that of ShuffleNetV2. From the perspective of lightweight design, SAFNet is the most compact model with only 0.73 M parameters and 0.46 M FLOPs, far smaller than other models such as ECA-Net (3.85 M parameters) and MobileNet.

In summary, SAFNet achieves an optimal balance between high classification accuracy, high efficiency, and lightweight design. It not only delivers top-level diagnostic performance but also offers the smallest model size and lowest computational complexity, proving its suitability for deployment on resource-constrained edge devices in marine engineering applications.

**Figure 9:**
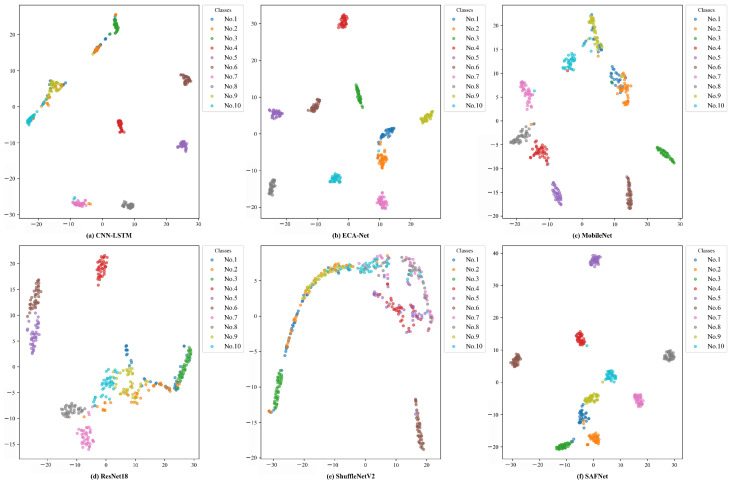
t-SNE distribution visualization of leakage features extracted by different models for marine oil and gas pipelines.

To compare the feature learning capabilities, we performed t-SNE feature visualization analysis under the low-pressure operating condition of 2 MPa. Analysis of Figure 9 reveals the excellent feature distribution characteristics of SAFNet. SAFNet exhibits superior feature discrimination capability: the valve leakage samples in the feature space show clear clustering patterns, with samples from all 10 leakage categories forming highly compact clusters. There are no obvious scattered or elongated samples, resulting in high intra-class cohesion and clear inter-class boundaries, with only a tiny number of overlapping samples.

In contrast, the second-best method, ECA-Net, shows good feature discrimination but exhibits significant local sample overlap. For MobileNet and CNN-LSTM, while samples of some categories form preliminary clusters, they still suffer from elongation and dispersion, indicating insufficient intra-class consistency and obvious sample overlap. ResNet18 and ShuffleNetV2 show severe mixing of samples from different categories, with blurred boundaries and even complete overlap, leading to weak feature separability and potential classification confusion. These findings further verify the excellent feature extraction capability of SAFNet under complex operating conditions.

**Figure 10:**
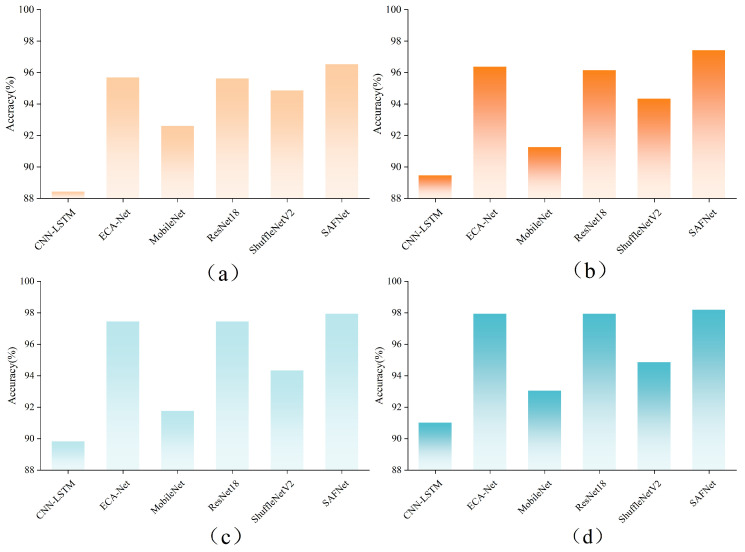
Classification accuracy comparison between SAFNet and mainstream models under different pressure conditions. (**a**) 2 MPa; (**b**) 3 MPa; (**c**) 4 MPa; (**d**) 5 MPa.

Figure 10 presents the comparative performance of various models for marine oil and gas pipeline valve leak localization under internal pressures ranging from 2 to 5 MPa. It can be noted that SAFNet achieves the highest accuracy under all four pressure conditions (2 MPa, 3 MPa, 4 MPa, and 5 MPa), with a consistent lead over the second-best models (ECA-Net or ResNet18) of approximately 0.5–1.15%. Compared with the lowest-performing CNN-LSTM, SAFNet outperforms it by 8.0–9.5 percentage points, representing a significant accuracy improvement. Furthermore, as the pressure increases from 2 MPa to 5 MPa, the accuracy of SAFNet remains consistently between 96.5% and 98.5%, with minimal fluctuation. This indicates that SAFNet can stably adapt to leakage signal characteristics under different pressures, unaffected by changes in operating conditions, and exhibits far superior robustness compared to other models.

Combined with previous experimental results, while maintaining the highest accuracy under all operating conditions, SAFNet also boasts the advantages of lightweight design, low computational complexity, high inference speed, and excellent feature extraction capability. It achieves the optimal balance among strong robustness, high efficiency, and high accuracy—something other models cannot achieve simultaneously. This means that SAFNet enables accurate leak identification within the common pressure range of marine oil and gas pipelines, covering scenarios from low-pressure micro-leakage to high-pressure severe leakage. Its stable performance avoids false positives or missed detections caused by varying operating conditions, improving the reliability of the monitoring system. Its lightweight and high efficiency make it easier to deploy on resource-constrained edge terminals, meeting the long-term unattended monitoring needs of offshore sites. Thus, SAFNet provides a feasible industrial technical solution for three major challenges in marine oil and gas pipeline monitoring.

### 4.5. Robustness Experiment Analysis

To comprehensively evaluate the robustness of SAFNet under various noise conditions, Gaussian white noise with signal-to-noise ratios (SNR) ranging from 0 dB to 20 dB, as well as impulsive noise with a proportion of 0.01 and an intensity factor of 10, were introduced into the dataset under the condition of 5 MPa. Specifically, the performance of SAFNet and comparison models was examined under multiple SNR conditions including 0 dB, 5 dB, 10 dB, 15 dB, and 20 dB.

**Figure 11:**
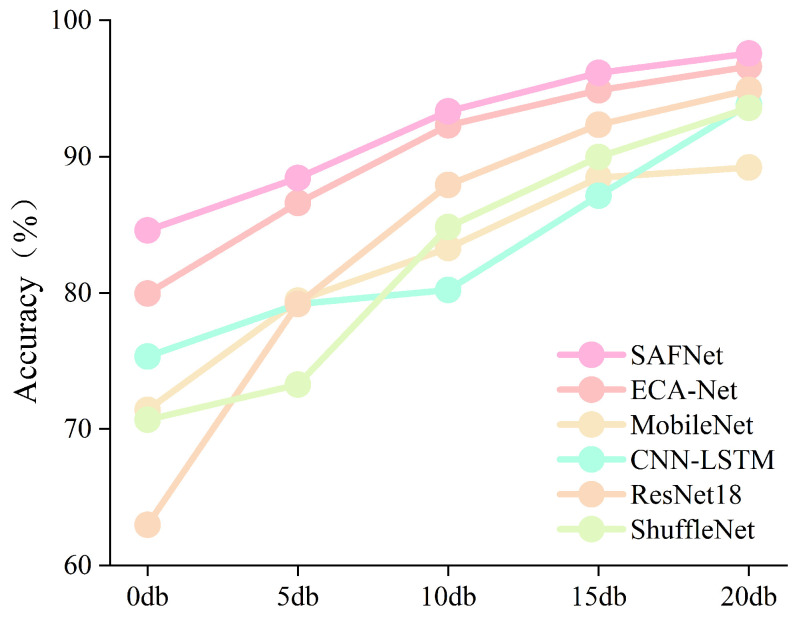
Anti-noise performance comparison between SAFNet and comparison models under multiple noise intensities.

As shown in the Figure 11, this study compares the classification performance of SAFNet with five mainstream baseline models (i.e., ECA-Net, ResNet18, MobileNet, CNN-LSTM, and ShuffleNet) under signal-to-noise ratios ranging from 0 dB to 20 dB. The results demonstrate that SAFNet maintains the highest classification accuracy across the entire noise intensity range. Specifically, SAFNet achieves an accuracy of 84.8% at an ultra-low signal-to-noise ratio of 0 dB, and its performance increases steadily with an improved signal-to-noise ratio, exceeding 97.5% at 20 dB. In contrast, all other baseline models suffer from significant performance degradation under low signal-to-noise ratio conditions. ResNet18 only achieves an accuracy of 63% at 0 dB, while models such as ShuffleNet and CNN-LSTM yield accuracies below 75%. Even under high signal-to-noise ratio conditions, the accuracy of most baseline models remains below 95%. These results fully verify that SAFNet can effectively decouple leakage features from marine noise, stably extract discriminative features even in strong noise environments, and exhibits significantly superior anti-noise robustness compared with all comparative models.

### 4.6. Ablation Study Analysis

To systematically verify the necessity and effectiveness of each core component of the proposed lightweight star-shaped attention-guided fusion network (SAFNet) for subsea pipeline leak detection based on acoustic emission (AE) signals, stratified ablation experiments are conducted under three representative working conditions, namely clean AE signals collected at 5 MPa operating pressure, clean AE signals collected at 2 MPa operating pressure, and low-SNR AE signals with 5 dB noise interference under 5 MPa. The complete SAFNet is regarded as the baseline model, and each ablation variant is constructed by removing one individual core module at a time. All model variants adopt identical training hyperparameters, fixed train-test data partitioning rules and unified hardware testing environments. Two core evaluation metrics including classification accuracy and weighted F1-score are utilized for comprehensive quantitative assessment, and all experimental results are summarized in Table 6.

Table 6 demonstrates that all core components are crucial to the performance of SAFNet. First, across all three experimental conditions, removing the PLSA module causes dramatic degradation of diagnostic performance. Under the clean 5 MPa dataset, the classification accuracy and weighted F1-score decrease by 11.05 and 15.18 percentage points, respectively, while reductions of 10.54 and 14.22 percentage points are observed under 5 dB low-SNR interference. The TPE module provides indispensable multi-granularity temporal modeling capability, and its removal leads to accuracy declines of 9.25%, 22.85%, and 14.65% across the three test scenarios. In comparison, eliminating the MS-GAF module only results in minor accuracy drops of 1.05%, 2.29%, and 2.57%. These distinct performance differences demonstrate that TPE and PLSA collectively form the fundamental feature extraction backbone of SAFNet. TPE captures multi-scale temporal pulse features to adapt to pressure-induced variations in leakage pulse width, while PLSA adaptively screens sparse leakage shock pulses and suppresses stationary background noise. The absence of either module removes the core physical leakage signatures required for diagnosis, leading to severe accuracy deterioration across all working conditions. Differing from the backbone modules, MS-GAF acts as a lightweight post-processing fusion optimizer. It aligns local fine-grained transient features with global long-range temporal representations to narrow inter-class feature distribution gaps and mitigate residual noise interference. Since the TPE–PLSA backbone can independently implement reliable fault identification via inherent leakage characteristics, removing MS-GAF only induces limited performance loss.

Secondly, the stratified multi-condition ablation data quantitatively reveals differentiated contribution weights of each core module under variable pressure operating regimes and low-SNR offshore environments. The PLSA module carries the highest contribution weight under low-SNR interference scenarios. When the operating pressure reduces from 5 MPa to 2 MPa, the magnitude of performance degradation after removing TPE expands sharply. Low-SNR interference further amplifies the performance gain brought by MS-GAF: the accuracy gap between the full SAFNet baseline and the variant without MS-GAF widens from 1.05% under clean 5 MPa signals to 2.57% under 5 dB noise contamination. This phenomenon arises because cross-scale feature aggregation implemented by MS-GAF effectively converges scattered leakage feature clusters distorted by intense offshore environmental noise.

Finally, the auxiliary modules DBSM and EDFR stably yield consistent performance improvements across all tested pressure and SNR conditions. Their contribution weights do not fluctuate drastically with varying working conditions, enabling sustainable secondary feature refinement without condition-dependent performance failure.

In summary, all core components of SAFNet produce synergistic effects to achieve robust subsea pipeline leakage diagnosis. The TPE-PLSA backbone undertakes fundamental feature extraction tasks, while MS-GAF, DBSM and EDFR serve as lightweight supplementary optimization modules. The differentiated contribution weight of each module under diverse working conditions further verifies the rationality of the hierarchical architecture proposed in this paper, and highlights the outstanding anti-noise capacity and cross-pressure generalization performance of SAFNet for practical offshore industrial monitoring applications.

The choice of designating one channel as the star center is motivated by the physical propagation characteristics of AE signals in pipelines. Leak-induced AE signals typically exhibit a coherent wavefront that arrives at the sensor with consistent phase and amplitude modulation across channels (the ‘core’ signature), while ambient noise and reverberations appear as incoherent, channel-specific perturbations (the ‘peripheral’ components). The star topology explicitly models this center-periphery structure, enabling the network to separate coherent leak signals from incoherent noise without learnable parameters. To validate the physical interpretability of the star-topology design in the DBSM module and to assess its sensitivity to the choice of the central feature channel, we conducted an additional ablation experiment by varying the strategy for selecting the star center from the multi-channel feature map. eight selection strategies were compared: (1) default, (2) back-half (the latter half of channels), (3) even (even-indexed channels), (4) odd (odd-indexed channels), (5) first-third (the first one-third of channels), (6) middle-third (the middle one-third), and (7–8) random_1 and random_2 (two independent random selections).

**Figure 12:**
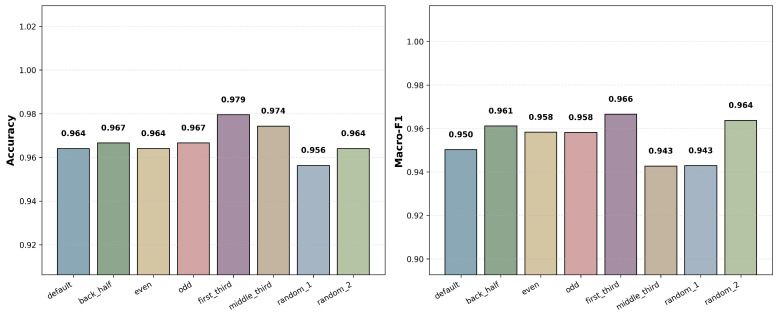
Performance Ablation of Different Star Center-Channel Selection Strategies for the DBSM Module.

As illustrated in Figure 12, the first_third strategy achieves the optimal performance among all candidate schemes, with a classification accuracy of 0.979 and a macro-F1 score of 0.966. The back_half and random_2 schemes deliver moderate performance and form the second tier, while the two random sampling strategies (random_1 and random_2) yield the lowest accuracy and F1 scores. It is worth noting that all fixed channel selection strategies can produce stable and outstanding diagnostic results, and the performance gap between the default scheme and the optimal first_third configuration is minor. The overall performance difference across all schemes is merely 3.6 percentage points. This experiment verifies that the star-topology feature aggregation mechanism within the DBSM module is robust to the selection of central feature channels and can adapt to various channel division strategies.

### 4.7. Interpret-Ability Analysis

**Figure 13:**
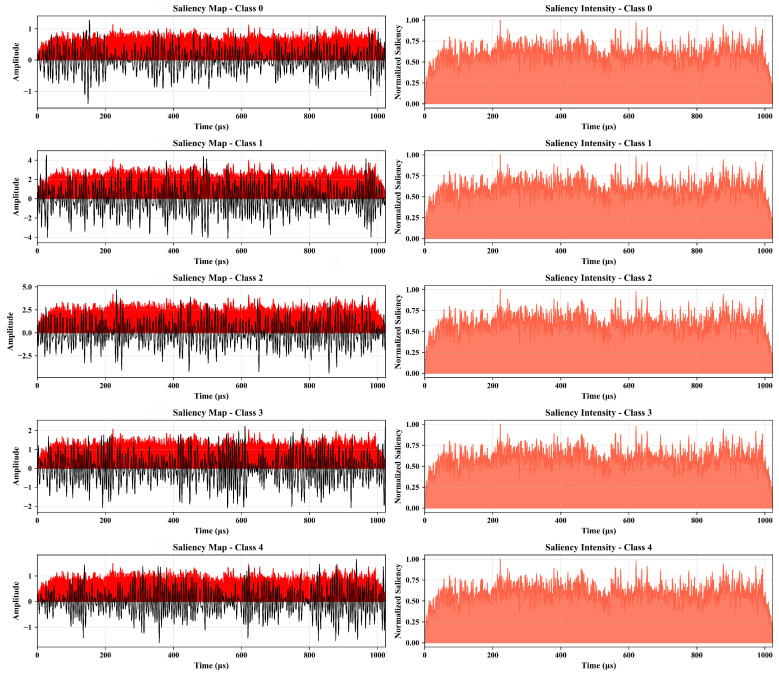
Temporal saliency analysis for leakage signals of Valve No. 0 to Valve No. 4 under 5 MPa pressure.The left column presents saliency maps with black curves for raw signal amplitude and red regions marking salient signal segments. The right column displays normalized saliency intensity filled with orange.

**Figure 14:**
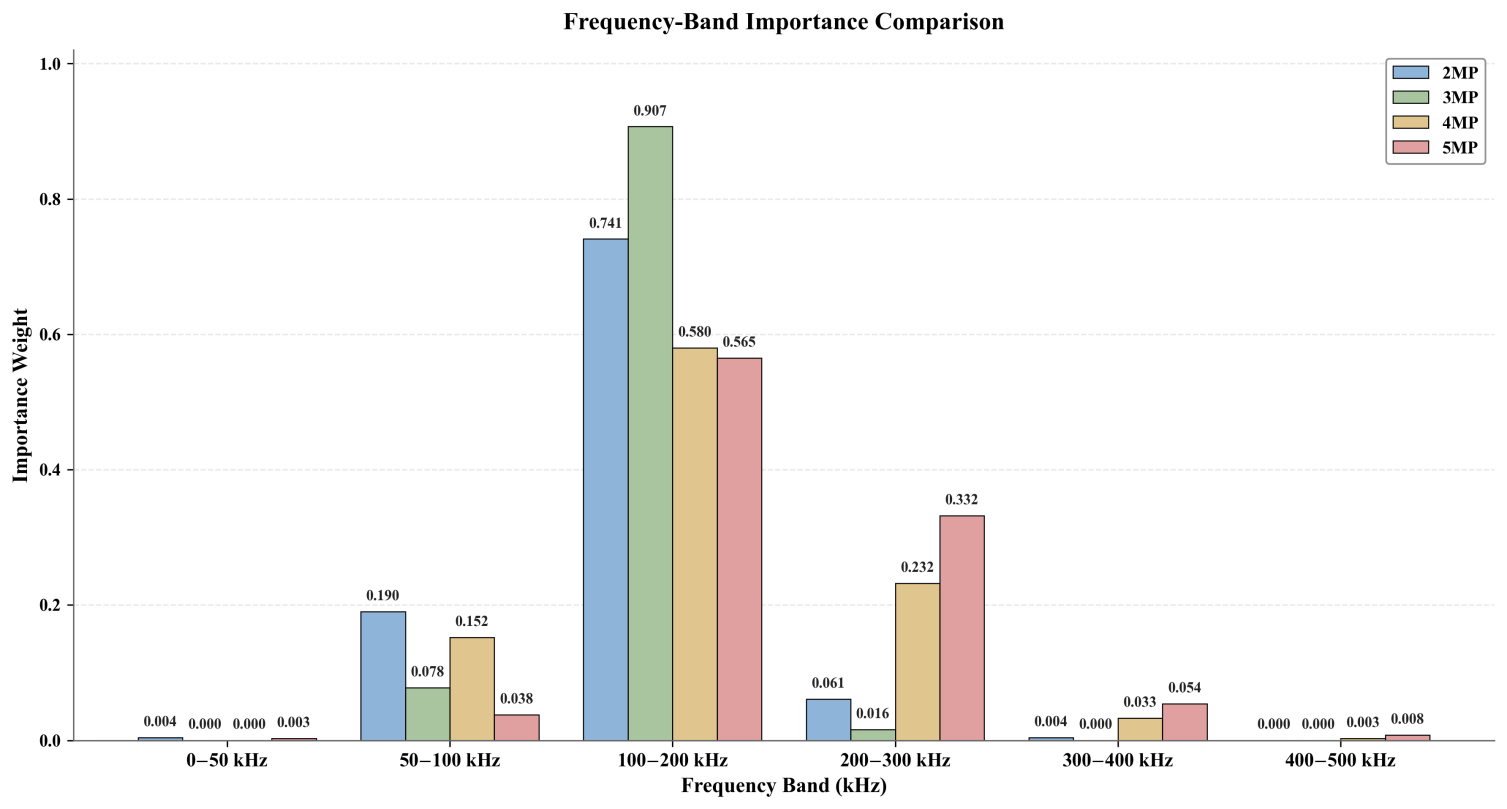
Normalized importance weights of six frequency bands under 2 MPa, 3 MPa, 4 MPa and 5 MPa operating pressures.

To explore the internal feature extraction mechanism of the proposed SAFNet and explain its remarkable generalization performance under multiple working conditions, two interpretability analyses including temporal saliency visualization and frequency-band importance statistics are carried out based on acoustic emission signals collected from leaking valves. Firstly, Figure 13 presents the temporal saliency analysis results under 5 MPa operating pressure, which consists of five groups of subplots corresponding to leakage conditions of Valve No. 0 to Valve No. 4. It can be clearly observed that although the amplitude of leakage signals collected from different valves varies significantly, the proposed model can consistently focus on the transient shock pulses induced by fluid leakage for all five valve samples, while suppressing steady background noise automatically. This phenomenon demonstrates that the network learns universal temporal leakage signatures rather than waveform patterns exclusive to a specific valve. Secondly, Figure 14 further reveals the inherent frequency preference of SAFNet by counting the feature importance of each frequency band under four operating pressures of 2 MPa, 3 MPa, 4 MPa and 5 MPa. The 100–200 kHz band acts as the dominant feature interval for valve identification, with a maximum normalized importance weight of 0.907, while the 200–300 kHz band serves as the auxiliary feature source. On the contrary, frequency bands below 100 kHz and above 300 kHz provide nearly negligible diagnostic information under all pressure conditions. Notably, the relative ranking of frequency band importance remains stable regardless of pressure fluctuations, which indicates that the attention module can extract pressure-robust high-frequency leakage features within the 100–300 kHz range. The temporal and frequency-domain visualization results fundamentally illustrate the outstanding cross-pressure generalization ability of SAFNet: the network only captures physically invariant inherent leakage features independent of variable working conditions, and filters out irrelevant noise components susceptible to environmental disturbances.

### 4.8. Generalization Evaluation

To verify the generalization capability of the proposed SAFNet, supplementary generalization tests are carried out on Dataset B, a public pipeline monitoring benchmark published by the University of Texas at Austin [61]. The dataset comprises pipeline networks with both loop and branched topologies, annotated with five distinct operational conditions: hole leakage, longitudinal cracks, circumferential cracks, gasket failure, and intact normal operation. All signals in Dataset B are collected using accelerometers, which capture vibration signatures triggered by fluid leakage originating from diverse structural defects on pipe walls, instead of only recording signals generated by internal valve leakage.Detailed partitioning specifications are presented in Table 7.

**Figure 15:**
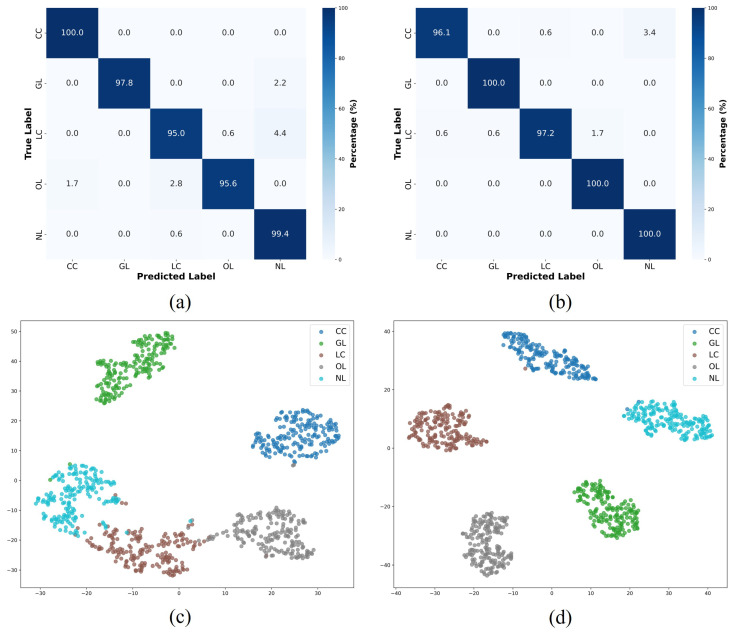
Classification and feature visualization results of SAFNet on Dataset B with two pipeline topological layouts. (**a**,**c**) Branched pipeline; (**b**,**d**) Looped pipeline.

Figure 15 presents the classification results and feature embedding visualizations of SAFNet under two pipeline structures. Subplots (a) and (c) correspond to the branched pipeline condition, achieving an overall test accuracy of 0.9755; Subplots (b) and (d) show results for the looped pipeline layout, with a higher overall accuracy of 0.9866. As observed from the confusion matrices, SAFNet maintains high per-class recognition accuracy above 95% for all five fault categories under both topological scenarios, with only tiny inter-class misclassification occurring between similar structural defects. The corresponding t-SNE feature distributions further demonstrate that the feature representations learned by SAFNet form compact, well-isolated clusters without severe cross-category overlap, which verifies the powerful discriminative capacity of the proposed star-shaped attention modules.

The stable high-accuracy performance on both branched and looped pipeline layouts, combined with reliable identification of various pipeline wall damage modes, sufficiently proves that SAFNet is not a task-specific diagnostic model limited to offshore oil and gas valve leakage monitoring. The multi-scale temporal pyramid encoder and parameter-free star-shaped attention mechanism can extract universal transient leakage-induced fluctuation features from arbitrary one-dimensional time-series signals, delivering strong generalization capability for diverse pipeline leakage diagnosis tasks across different working conditions, sensing modalities and fault types.

### 4.9. Edge Deployment Performance Evaluation

**Figure 16:**
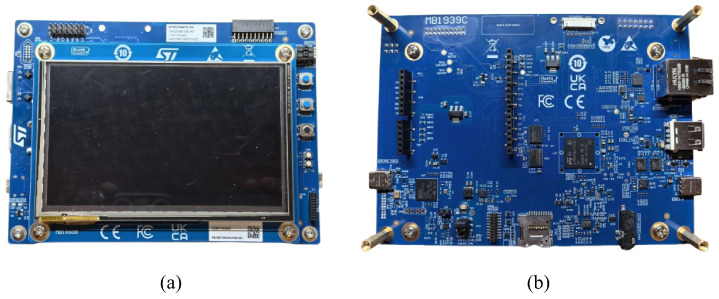
Physical photo of the STM32N657X0H3Q evaluation board. (**a**) Front view with touch LCD screen; (**b**) Rear view showing the main circuit layout and peripheral interfaces.

To further verify the practical engineering applicability and edge deployment capability of the proposed SAFNet, real embedded deployment tests are conducted on industrial-grade ultra-low-power microcontroller units (MCU). In this work, the STM32N657X0H3Q chip is selected as the target edge hardware platform, which is equipped with an ARM Cortex-M55 core and a dedicated ST Neural-ART hardware accelerator, fully meeting the real-time inference requirements of offshore field monitoring terminals. The physical appearance of the evaluation board is presented in Figure 16 and the detailed hardware specifications of the adopted MCU are summarized in Table 8.

In this embedded experiment, SAFNet is deployed in full FP32 floating-point precision. To ensure credible and stable inference results, the model inference speed is tested repeatedly for ten independent runs. The recorded inference time fluctuates slightly within a narrow range, with an average inference latency of 14.948 ms per sample, a minimum speed of 14.174 ms, and a maximum speed of 16.240 ms. The comprehensive edge deployment resource consumption and inference performance are listed in Table 9.

Notably, all experiments adopt full FP32 precision deployment rather than INT8 quantization. Since the STM32N657X0H3Q platform provides complete hardware floating-point computation support and sufficient on-chip memory resources, SAFNet can run stably and efficiently under original FP32 precision. Therefore, additional quantization compression is unnecessary, which effectively avoids the accuracy loss caused by low-bit quantization and maintains the optimal diagnostic performance of the model in actual edge deployment scenarios.

In general, SAFNet exhibits extremely low weight storage overhead, negligible activation memory occupation, and stable millisecond-level inference speed on embedded ARM edge devices. The superior lightweight and real-time performance further demonstrate the great potential of SAFNet for practical field deployment in offshore pipeline intelligent monitoring systems.

## 5. Discussion and Limitations

While the proposed SAFNet demonstrates compelling performance in terms of accuracy, robustness, and computational efficiency, it is imperative to contextualize these achievements within the explicit boundaries of our experimental design and to discuss the factors that may influence its real-world deployment.

This work solely focuses on the detection and spatial localization of internal leakage at pipeline valves. Comprehensive subsea pipeline integrity management demands an integrated diagnostic framework capable of identifying a full spectrum of progressive failure modes, including incipient crack propagation, corrosion-induced wall thinning, and fatigue degradation. SAFNet is engineered exclusively as a dedicated front-end module for rapid valve leakage alerting, rather than a universal diagnostic solution covering all types of structural defects. A primary direction for follow-up research lies in extending the proposed star-shaped attention architecture to simultaneously discriminate leakage, crack, and corrosion signals. Meanwhile, further mechanistic investigations will be conducted to interpret the evolution of structural damage and enable early warning before leakage initiates.

Secondly, a central limitation of the current study lies in our data acquisition strategy. Offshore valve internal leakage constitutes a low-probability yet highly destructive failure mode. Fixed offshore production platforms enforce strict safety and marine environmental protection protocols that prohibit intentional artificial leakage trials, rendering large-scale collection of labeled natural leakage signals practically infeasible. The leakage-related acoustic emission signatures were therefore obtained from controlled laboratory experiments under well-defined pressure gradients (2–5 MPa) and valve configurations, while the background noise was independently recorded from the Liwan 3-1 offshore platform. These two signal sources were subsequently combined via linear superposition to construct the training and test sets. This linear signal mixing workflow is not an ad-hoc makeshift solution; it has been widely adopted and experimentally validated as a standardized data construction paradigm across multiple engineering disciplines outside subsea pipeline monitoring, including aerospace composite damage detection [62], rotating machinery bearing fault diagnosis [63], and civil bridge structural health monitoring with synthetic superimposed interference signals [64]. We explicitly acknowledge that this linear mixing model represents a simplified approximation of reality. In actual offshore environments, the interaction between leakage-induced stress waves and ambient oceanic noise (including wave impacts, machinery vibrations, and flow-induced turbulence) is inherently nonlinear, involving amplitude modulation, frequency intermodulation, and time-varying channel effects. Consequently, the fusion dataset carries distinct methodological merits despite its simplification: it delivers fully reproducible samples with precisely controllable SNR gradients, forming a fair, standardized benchmark for objective algorithm comparison, yet it does not fully replicate the complex physical coupling present in a genuine subsea leakage incident. We therefore restrict all performance claims of SAFNet strictly to this linearly simulated acoustic setting and emphasize that transfer learning or domain adaptation trained on authentic offshore leakage recordings will be indispensable for bridging the simulation-to-reality generalization gap in future on-site deployments.

## 6. Conclusions

To address the practical engineering challenges in offshore oil and gas pipeline valve leak detection, including redundant parameters, high inference latency, low distinguishability of weak leakage features under strong marine noise, and poor deployability on edge devices, this study proposes a Parameter-free Star-shaped Attention Fusion Network (SAFNet) that establishes an end-to-end lightweight leak diagnosis framework for industrial process safety and marine environmental protection. SAFNet is built with a Temporal Pyramid Encoder (TPE) and three-stage progressive lightweight Star-shaped Attention (PLSA) as the backbone. It integrates Bilinear Star-shaped Mapping (DBSM), Energy-driven Feature Refiner (EDFR), and Multi-scale Gated Attention Fusion (MS-GAF) to achieve efficient representation of acoustic emission leakage signals through multi-scale feature extraction, parameter-free enhancement, noise-resistant refinement, and adaptive fusion, thereby breaking the critical bottleneck where weak leakage signals are submerged in strong marine noise. Experiments show that SAFNet has only 0.73 M parameters, 0.46 M FLOPs, and a single-sample inference time of 313.81 μs. Its accuracy remains stable between 96.5% and 98.5% under working pressures of 2–5 MPa, respectively, significantly outperforming mainstream comparison models. Combining high accuracy, lightweight design, strong anti-noise capability, and low latency, SAFNet meets the requirements of edge deployment and real-time monitoring. Hyperparameter analysis, t-SNE feature visualization, ablation experiments, and robustness tests further validate the performance and module effectiveness of SAFNet. This research provides a lightweight intelligent diagnosis solution for marine oil and gas pipeline leak detection. Its parameter-free star-shaped attention and multi-scale fusion strategy offers new insights into the structural health monitoring of offshore platform pipelines, holding important engineering value and theoretical significance for ensuring the process safety of marine oil and gas engineering and preventing marine environmental pollution.

## Figures and Tables

**Table 1:** Symbol definitions and dimensions for the SAFNet model.

Symbol	Physical Meaning	Dimension
*X*	Acoustic Emission signals	$R^{B\times L}$
$F_{in}$	Module Input Feature Map	$R^{B\times C\times L}$
$F_{out}$	Module Output Feature Map	$R^{B\times C^{\prime }\times L^{\prime }}$
$F_{c}$	Key Features of a Star-Shaped Structure	$R^{B\times 1\times L}$
$F_{p,i}$	The *i*-th outer feature of the star-shaped structure	$R^{B\times 1\times L}$
*k*	Convolution kernel size	scalar
$k_{c}$	Channel-specific convolution kernels	one-dimensional vector
*s*	Convolution stride	scalar
*p*	Number of convolution strides	scalar
∗	One-dimensional convolution operation	-
⊙	Hadamard product	-
$W_{c},W_{p}$	Bicubic interpolation with a fixed weight matrix	$R^{L\times L}$
$W_{fc},b_{fc}$	Decision-maker linear layer parameters	$R^{C_{\mathrm{final}}\times N},R^{N}$
$\mu_{c}$	Mean of the feature channel	scalar
$\sigma_{c}$	Global average pooling	scalar
$S_{c}$	Energy importance score	scalar
ϵ	Regularization constant	scalar
$b_{0}$	Convolution with a fixed bias	scalar
$N_{\mathrm{PLSA}}$	Number of stacked PLSA modules	scalar
*P*	Probability of failure prediction	$R^{B\times N}$

**Table 2:** Distribution positions of the valves relative to the sensor.

Valve No.	Position (mm)
1	207
2	567
3	807
4	1253
5	1923
6	2406
7	2895
8	3500
9	4105
10	4397

**Table 3:** Partitioning details of the acoustic emission (AE) Dataset.

Scenario	Label	Training Set	Test Set
Valve No. 1	0	156	39
Valve No. 2	1	156	39
Valve No. 3	2	156	39
Valve No. 4	3	156	39
Valve No. 5	4	156	39
Valve No. 6	5	156	39
Valve No. 7	6	156	39
Valve No. 8	7	156	39
Valve No. 9	8	156	39
Valve No. 10	9	156	39

**Table 4:** Performance comparison between PLSA and representative learnable attention mechanisms.

Variant	Attention Mechanism	Params (M)	Inference (ms)	ACC (%)	F1 (%)
A (SAFNet)	PLSA	0.7255	2.604	98.200	98.196
B	SE	0.7358	2.763	97.943	97.914
C	ECA	0.7255	2.992	98.458	98.452
D	CBAM	0.7358	3.703	98.200	98.196

**Table 5:** Performance comparison between SAFNet and mainstream deep learning models.

Index	CNN-LSTM	ECA-Net	MobileNet	ResNet18	ShuffleNetV2	SAFNet
Average ACC (%)	75.84	97.69	95.12	96.14	95.63	97.69
F1 (%)	72.04	97.68	95.08	96.13	95.60	97.68
Training time (s)	71.43	168.54	165.68	157.31	149.43	72.69
Inference time (μs)	54.12	354.21	378.65	344.83	509.94	313.81
Params (M)	0.98	3.85	3.71	3.85	1.25	0.73
FLOPs (M)	50.65	1.51	2.78	1.49	1.00	0.46

**Table 6:** Stratified ablation results of SAFNet under multiple offshore operating conditions.

Model Configuration	5 MPa		2 MPa		5 MPa (5 dB SNR)	
	Accuracy	F1-Score	Accuracy	F1-Score	Accuracy	F1-Score
Full SAFNet (Baseline)	0.98194	0.98108	0.96116	0.96112	0.89974	0.89821
w/o TPE	0.88946	0.88715	0.73265	0.69592	0.75321	0.71952
w/o DBSM	0.96401	0.96388	0.94602	0.94589	0.83033	0.82597
w/o EDFR	0.96603	0.96541	0.95887	0.95879	0.87918	0.87776
w/o PLSA	0.87147	0.82933	0.89974	0.89929	0.79434	0.75603
w/o MS-GAF	0.97143	0.97111	0.93830	0.93842	0.87404	0.87068

**Table 7:** Partitioning details of Dataset B.

Scenario	Label	Training Sets	Testing Sets
Looped–Circumferential Crack (CC)	0	718	180
Looped–Gasket Leak (GL)	1	718	180
Looped–Longitudinal Crack (LC)	2	718	180
Looped–Orifice Leak (OL)	3	718	180
Looped–No-Leak (NL)	4	718	180
Branched–Circumferential Crack (CC)	0	718	180
Branched–Gasket Leak (GL)	1	718	180
Branched–Longitudinal Crack (LC)	2	718	180
Branched–Orifice Leak (OL)	3	718	180
Branched–No-Leak (NL)	4	718	180

**Table 8:** Technical Specifications of STM32N657X0H3Q Edge MCU Platform.

Parameter	Specification
MCU Model	STM32N657X0H3Q
CPU Core	32-bit ARM Cortex-M55
Maximum CPU Frequency	800 MHz
Embedded SRAM	4.2 MB contiguous SRAM
External Flash	1-Gbit Octo-SPI Flash Memory
Floating-Point Unit	Support for IEEE 754 half/single/double precision
Hardware Neural Accelerator	ST Neural-ART, up to 1 GHz, 600 GOPS peak throughput
Operating Voltage	1.71 V∼3.6 V
Operating Temperature	−40 °C∼+125 °C

**Table 9:** Edge Deployment Resource Consumption and Inference Performance of SAFNet.

Performance Index	Value
Total Parameters	732,937 (2.80 MiB)
Total MACCs	32,827,064
Flash Memory Occupation (Weight)	2.80 MiB
RAM Activation Memory	73.89 KiB
Average Inference Latency	14.948 ms/sample
Minimum Inference Latency	14.174 ms/sample
Maximum Inference Latency	16.240 ms/sample
Deployment Precision	Full FP32 floating-point

## Data Availability

Data available on request from the corresponding author.
